# Development of a phenotyping platform for high throughput screening of nodal root angle in sorghum

**DOI:** 10.1186/s13007-017-0206-2

**Published:** 2017-07-11

**Authors:** Dinesh C. Joshi, Vijaya Singh, Colleen Hunt, Emma Mace, Erik van Oosterom, Richard Sulman, David Jordan, Graeme Hammer

**Affiliations:** 1 ICAR- Indian Grassland and Fodder Research Institute, Jhansi, Uttar Pradesh 284003 India; 2 Queensland Alliance for Agriculture and Food Innovation, The University of Queensland, Brisbane, QLD 4072 Australia; 3 Department of Agriculture and Fisheries, Hermitage Research Facility, Warwick, QLD 4370 Australia; 4 Biosystems Engineering, 323 Margaret Street, Toowoomba, QLD 4350 Australia; 5 Queensland Alliance for Agriculture and Food Innovation, Hermitage Research Facility, The University of Queensland, Warwick, QLD 4370 Australia

**Keywords:** Drought, High throughput phenotyping, Root system architecture, Nodal root angle, Sorghum

## Abstract

**Background:**

In sorghum, the growth angle of nodal roots is a major component of root system architecture. It strongly influences the spatial distribution of roots of mature plants in the soil profile, which can impact drought adaptation. However, selection for nodal root angle in sorghum breeding programs has been restricted by the absence of a suitable high throughput phenotyping platform. The aim of this study was to develop a phenotyping platform for the rapid, non-destructive and digital measurement of nodal root angle of sorghum at the seedling stage.

**Results:**

The phenotyping platform comprises of 500 soil filled root chambers (50 × 45 × 0.3 cm in size), made of transparent perspex sheets that were placed in metal tubs and covered with polycarbonate sheets. Around 3 weeks after sowing, once the first flush of nodal roots was visible, roots were imaged in situ using an imaging box that included two digital cameras that were remotely controlled by two android tablets. Free software (*openGelPhoto.tcl*) allowed precise measurement of nodal root angle from the digital images. The reliability and efficiency of the platform was evaluated by screening a large nested association mapping population of sorghum and a set of hybrids in six independent experimental runs that included up to 500 plants each. The platform revealed extensive genetic variation and high heritability (repeatability) for nodal root angle. High genetic correlations and consistent ranking of genotypes across experimental runs confirmed the reproducibility of the platform.

**Conclusion:**

This low cost, high throughput root phenotyping platform requires no sophisticated equipment, is adaptable to most glasshouse environments and is well suited to dissect the genetic control of nodal root angle of sorghum. The platform is suitable for use in sorghum breeding programs aiming to improve drought adaptation through root system architecture manipulation.

## Background

Root system architecture (RSA) is a major factor determining the ability of plants to access soil moisture in drought prone environments, particularly in cereals like sorghum (*Sorghum bicolor* (L.) Moench), maize (*Zea mays* L.), and wheat (*Triticum aestivum* L.), which are frequently grown in such environments. Of the many traits constituting RSA, the growth angle of the seminal and nodal roots at the seedling stage has important implications for drought adaptation of adult cereal plants [1–3], because this trait can influence both horizontal and vertical exploration of the soil by roots [4–7]. These spatial effects on the ability of plants to access water can be exploited through crop management [8, 9].

The root system of sorghum is characterized by a single seminal root originating directly from the embryo and by multiple postembryonic nodal roots that emerge from the below-ground nodes of the stem [10].The seminal root plays an important part only in initial water and nutrient uptake and hence is of little importance in mature sorghum, for which the RSA is predominantly constituted by post embryonic nodal roots [11, 12]. The angle of the first flush of nodal roots, which appears when around five leaves have fully expanded [10], is associated with the spatial distribution of roots of mature sorghum plants and hence with their ability to extract soil water [7]. As a consequence, an association between nodal root angle and grain yield has been reported for sorghum [13]. Nodal root angle is thus an important selection trait in sorghum breeding programs for improving drought adaptation.

High throughput screening of agronomically relevant traits is often restricted by the availability of suitable phenotyping systems, rather than the availability of genetic information. Multiparental breeding populations such as nested association mapping (NAM) populations have emerged as an excellent mapping resource to dissect the genetic control of complex quantitative traits by combining the advantages of traditional linkage analysis with association mapping [14]. Genetic dissection of various complex agronomic traits has been investigated in maize by utilising abundant genetic diversity of a NAM population [15–22]. A comparable backcross (BC) nested association mapping (BC-NAM) population has been developed in sorghum [23] and has been used to dissect the genetic control of complex quantitative traits [24]. Despite the availability of mapping resources such as BC-NAM populations to tackle complex rooting traits, the lack of high throughput phenotyping methods remains a bottle neck to quantify the genetic control of nodal root angle and enable its use as a selection trait in breeding programs.

Various phenotyping methods have been proposed for screening of root traits, including root angle, in common bean (*Phaseolus vulgaris* L.) [25, 26], barley (*Hordeum vulgare* L. and *H. spontaneum* C. Koch) [1], wheat (*Triticum aestivum* L) [4], and maize (*Zea mays* L.) [2, 27]. However, all these methods are designed to measure root traits within a few days of germination and are not suitable for phenotyping of nodal roots in sorghum, which needs to grow for 3 weeks before the first flush of nodal roots starts to appear [10]. Field based phenotyping methods have been proposed for phenotyping postembryonic root architecture of adult plants [28–30]. Although these methods can provide good data on a range of traits associated with RSA, the throughput of this system is limited by the duration of the crop cycle, and the area of land required to screen large numbers of genotypes.

To exploit nodal root angle as a selection trait in sorghum breeding programs that target drought stressed environments, efforts need to be directed towards the development of robust root phenotyping platforms that are capable of (1) supporting root and shoot growth up to 5th–6th leaf stage, (2) expressing high heritability (repeatability) for the trait, and (3) minimizing the genotype × environment interaction. Ideally, any platform should have a short phenotyping cycle, be suitable for continuous screening throughout the year, and not be labour intensive. Therefore, the objectives of this study were to (1) develop a simple, low cost and high throughput phenotyping platform that supports root development of sorghum up to the 5th–6th leaf stage, (2) develop a high throughput imaging system for the digital measurement of nodal root angle and (3) test its high throughput capacity, reproducibility and ability to identify sorghum genotypes with contrasting root angle by characterizing a large BC-NAM population and a large set of advanced hybrids.

## Methods

### Phenotyping platform setup

As a first step in the development of a high throughput phenotyping platform for nodal root angle in sorghum, we compared the suitability of three artificial growing media, specifically gel chambers, seed germination blotting paper (Anchor Paper Co, St Paul, MN, USA), and geotextile capillary mat with pore size of 60 microns (Global Synthetics, Virginia, QLD, Australia), with soil filled chambers that were developed previously [10]. The gel filled observation chambers were constructed from two plates (one black perspex and one clear glass) as described by Bengough et al. [1] and Manschadi et al. [4]. Sterilized agar (Sigma Type A; 2% w/v) was poured onto each plate and after the agar had set, the two plates were taped together. Two germinated seeds were placed between the agar layers of the vertically mounted chambers. The seeds were oriented vertically with the radicle facing downwards. These gel chambers have been used successfully for wheat [4], where seminal root angle can be phenotyped within days of germination. However, they did not support the growth of sorghum plants for the extended period until the appearance of nodal roots, because of microbial contamination, difficulties in maintaining a consistent density of gel and an inability to meet the nutrient requirements of the plants.

The germination paper and capillary mat were cut into 40 × 35 cm units. Both were sterilized in an autoclave before being hung into a large plastic tub with similar height and width as the units so that the lowest 3 cm of the units were submerged in 3.5 l of nutrient solution, which moved up through the capillary mat and germination paper through capillary action. Both the germination paper and capillary mat were always wet throughout the experimental period, suggesting adequate capillary rise in both systems. The nutrient solution consisted of quarter strength of Hoagland solution and was changed every 5 days. Sorghum seeds were sterilized by rinsing in 70% ethanol and washed three times with sterile distilled water. The sterilized seeds were pre-germinated for 3 days on filter paper in petri dishes with a day/night temperature regime of 30/22 °C and 60% relative humidity. The germinated seeds were fixed between two sheets of germination paper or capillary mat with two standard paper clips and each unit was attached to a wooden stick with two fold back clips, one on each side of the upper edge of the unit. Each unit was covered with black polythene foil to prevent penetration of light and had one 2 × 1 cm slit at the top to allow emergence of the shoot.

The soil filled chambers, which consisted of two perspex sheets, separated by a rubber strip and clamped together with fold back clips, have been described in detail by Singh et al. [10]. The growth parameters of the plants grown in the soil-filled chambers were compared with those of plants grown on the germination paper and capillary mat. Shoot dry weight, root dry weight, root length, and root diameter of 3 week old plants were measured, when around five leaves had fully expanded and nodal roots started to appear. At harvest, the shoots of five plants from each of the three media were cut off at the base of the stem and shoot dry weight was determined after drying at 60 °C for 3 days. After imaging, roots were carefully removed from the medium and washed. Washed roots were stored in 70% ethanol and scanned using an Epson scanner (Epson, Long Beach, CA, USA), after submerging in a water bath to facilitate separation of roots and to minimize overlap. Scanning was done at a resolution of 600 dpi, using both top and bottom lighting and a threshold setting of 68 was used to distinguish roots from the background. Scanned roots were analyzed using WinRHIZO Pro (Regent Instruments, Inc., Quebec City, QC, Canada) which was calibrated to obtain total root length and average root diameter. Root samples were then dried at 60 °C for 3 days and dry weight determined.

**Fig. 1:**
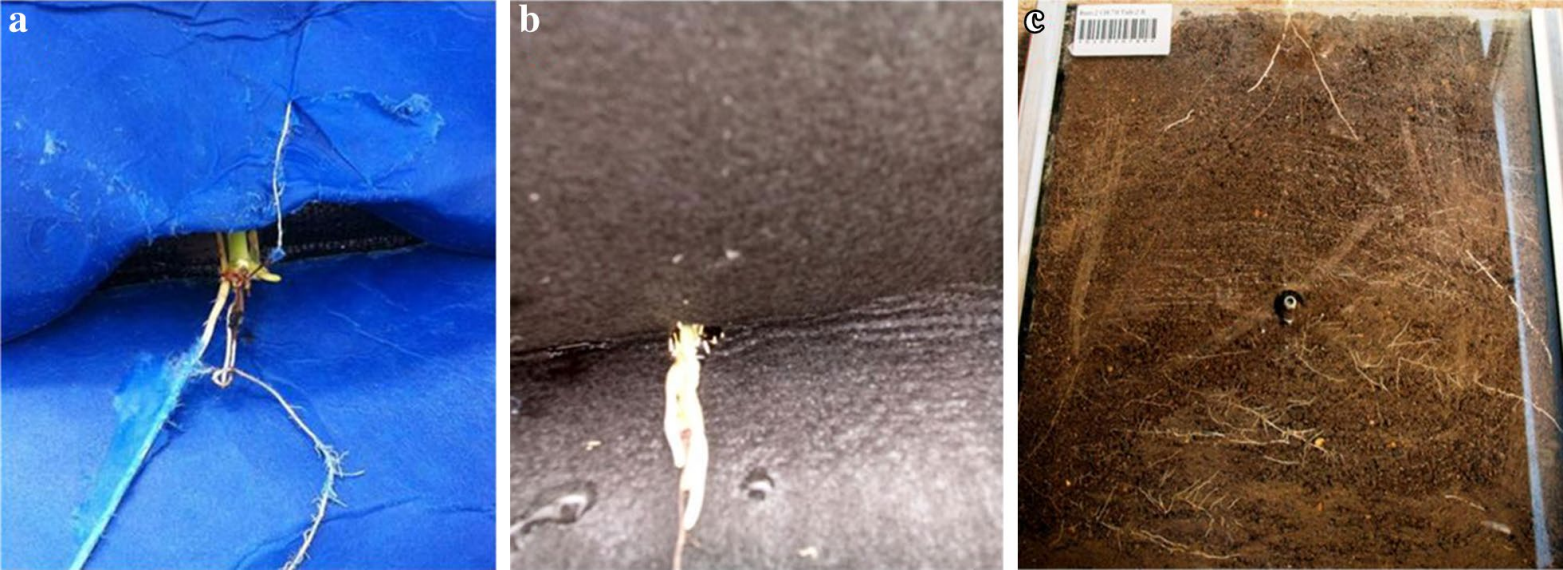
Close up of **a** roots growing into surface layers of the germination paper and **b** growing vertically on a capillary mat. In contrast, in soil-filled root chambers (**c**), well-developed nodal roots are clearly visible against the perspex sheet

The germination paper and capillary mat did support plant growth until five leaves had fully expanded, but plant growth was suboptimal and non-representative of soil grown plants. The rate of development was significantly slower than in soil filled chambers, as was evident from a slower leaf appearance rate and this delayed appearance of nodal roots at the five-leaf stage (Table 1). Despite this delayed harvest, plants had significantly shorter and thinner roots and less root and shoot biomass (Table 1). Moreover, nodal roots often penetrated the germination paper surface (Fig. 1a) or grew vertically along the capillary mat (Fig. 1b), making measurement of nodal root angle cumbersome or even impossible. In contrast, in the soil filled chambers, nodal roots became clearly visible against the transparent perspex sheets once they started appearing around the 5th leaf stage (Fig. 1c). The prolonged duration of the screens using germination paper and capillary mat made it more cumbersome to meet the nutrient requirements of the plants and to keep the surface of the growth medium free from microbial contamination. This required sterilization of the substrate and seeds, pre-germination of seeds, and preparation of Hoagland solution at regular intervals, making these two systems inefficient and more time consuming for screening large numbers of genotypes compared to the soil filled chambers. Hence, a phenotyping platform based on soil-filled chambers was deemed to be the most suitable for high throughput phenotyping of nodal root angle in sorghum.

**Table 1:** Comparison of seedling attributes using soil filled root chambers, germination paper and capillary mat

Trait	Soil filled chamber	Germination paper	*P* ^a^	Capillary mat	*P* ^b^
Days to emergence of nodal roots	25	36	0.001	36	0.001
Total root length (m)	312	245	0.003	232	0.002
Average root diameter (mm)	2	1.3	0.002	1.2	0.002
Root mass (g)	0.13	0.08	0.005	0.06	0.005
Shoot mass (g)	0.33	0.21	0.002	0.24	0.001

^a^ *P* value for difference of germination paper with soil filled chamber

^b^ *P* value for difference of capillary mat with soil filled chamber

### Soil based phenotyping platform

**Fig. 2:**
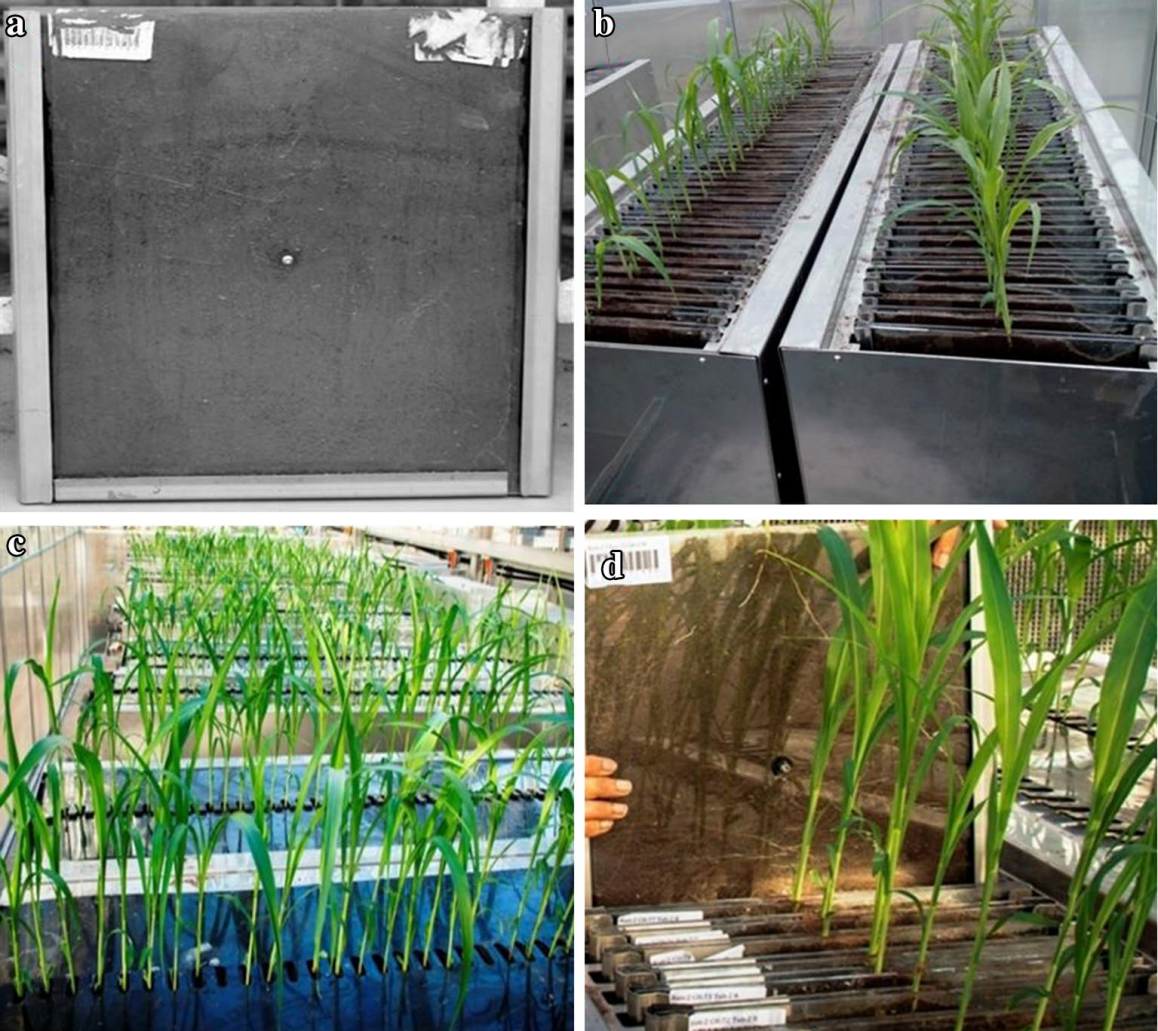
High throughput phenotyping platform for screening genetic variation for nodal root angle in sorghum. **a** Purpose built root chamber filled with soil. **b** Metal tubs containing root chambers. **c** Polycarbonate sheet covering the top of the root chambers to exclude light. **d** Nodal roots visible through the transparent wall of perspex sheets at 6th leaf stage

**Fig. 3:**
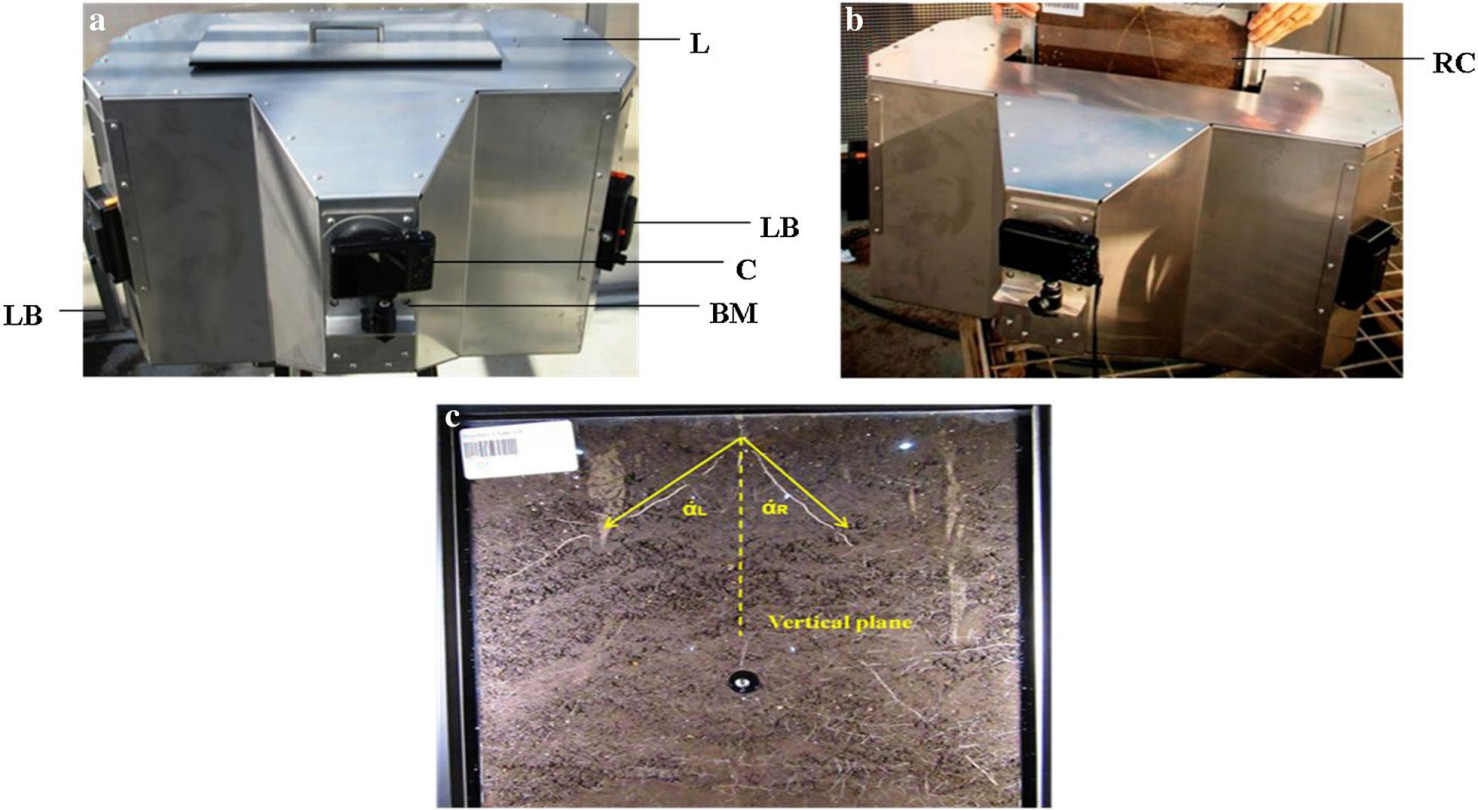
**a** High throughput imaging box with its components (C, camera; BM, ball mount; LB, light box; L, lid). **b** Imaging of nodal roots after harvest (RC, root chamber). **c** For each image, the left (αL) and the right (αR) angle between the first pair of nodal roots and the vertical plane was measured using the software package

The phenotyping platform consists of custom built root chambers (Fig. 2) and imaging equipment (Fig. 3) that were designed and constructed by Biosystems Engineering, Toowoomba, QLD, Australia (www.biosystem-eng.com). The platform was an improvement on a previously published system [31], which lacked automation for imaging and was not conducive for high throughput applications. The basic units of the phenotying platform were custom made root chambers (Fig. 2a). Each chamber comprised of two 6 mm thick transparent perspex sheets of 50 cm high, 45 cm wide and 3 mm thick that were separated on three sides (two long sides and one of the short sides) by 3 mm thick rubber and held in place by three metal clamps. To minimize bulging of the chambers during soil filling and to maintain constant 3 mm spacing between the two perspex sheets, the sheets were connected at the centre with a nut and bolt. Each chamber was filled with 1100 g of black, clay-textured soil, which provided a suitable contrast with the roots for image analysis. Root chambers were placed vertically in 2 m long stainless steel tubs that had similar height and width as the chambers (Fig. 2b). Each tub had slots at the top and bottom to vertically position 50 root chambers, and had six holes in the bottom to allow drainage of excess water and nutrient solution. The entire platform contained ten tubs, giving a total capacity of 500 root chambers.

Before sowing, the filled chambers were watered to field capacity. Three seeds were sown per chamber. In order to ensure root growth along the transparent perspex sheets, seeds were placed vertically at a depth of 3 cm, with the embryo downwards and facing one of the perspex sheets. After sowing, the top surfaces of all chambers in each tub were covered with 2 m long black polycarbonate sheets to exclude light from the developing roots while leaving 5 cm long slits for the seedlings to emerge (Fig. 2c). Three days after emergence, seedlings were thinned to one per chamber. A complete hydroponic nutrient solution (Peters Professional Water Soluble Fertilizer Hydro-sol, Scotts-Sierra Horticultural Products Co., Marysville, OH, USA) was applied once a week to ensure nutrients and water were not limiting growth and development of the plants. The solution comprised of the following nutrients (mg per liter of water); N, 200; P, 48; K, 210; Ca, 193; Mg, 40; Na, 3.6; S, 53.5; B, 0.50; Cl, 0.04; Cu, 0.15; Fe, 5.0; Mn, 0.50; Mo, 0.10; and Zn, 0.15.

Roots were imaged when 5–6 leaves had fully expanded and the first flush of nodal roots was visible. At that time, the shoot of each plant was removed by cutting the base of the stem. Previous studies have shown that differences in plant size and vigour do not affect root angle [31]. Both perspex sheets of each chamber were barcoded (Fig. 2d) to track the identity of individual plants during imaging. Root imaging was done in a metal box of 55 cm high, 62 cm wide, and 40 cm thick that contained a central imaging plane (Fig. 3). Two cameras (Canon PowerShot SX610 HS 20.2 MP Ultra-Zoom Digital Cameras) were positioned on versatile ball mounts (Universal 1/4”-20 Camera Accessory Mount) on either side of this plane (Fig. 3a). The imaging box also housed Glanz LED98A Camera LED light boxes (121 × 78 × 35 mm) that were mounted in four corners to uniformly illuminate the root chambers during imaging. These light boxes were fully dimmable and included one magnetic diffuser for soft day light balance light and a second one for the soft tungsten balance light. The imaging plane contained three metal clamps to allow easy insertion and removal of each root chamber, and to ensure a fixed position within the imaging plane. The cameras were equipped with in-built Wi-Fi^®^ technology to connect directly to Android™ devices (Samsung Galaxy Tab 3 Lite-7.0 T-113 Wifi-Only 8 GB White Tablets) using camera connect (www.canon.com.au), a free app that controlled the imaging set up and synchronized the imaging of both sides of each root chamber (Fig. 3b). An exposure time of 1 s allowed for high quality images with limited background noise (Fig. 3c). Hence, there was no need for calibrating the cameras. Root images, which captured the entire perspex sheets on both sides, were downloaded manually from the camera to a computer and were saved as JPEG files.

### Plant material

Two different sets of sorghum germplasm were used in the study: a backcross nested association mapping (BC-NAM) population and a set of advanced hybrids from the Australian sorghum pre-breeding program. The BC-NAM population was derived from crosses between a recurrent inbred line (R931945-2-2) and 23 diverse founder parents [23]. R931945-2-2 is an elite line adapted to Australian growing environments and the founder parents include exotic genotypes that represent the global diversity of sorghum, spanning countries of origin, racial type, and wild and weedy genotypes. In total, 976 BC-NAM progenies and 24 parents were included in the study. The advanced hybrid set included 628 hybrids that were based on three female and 395 male parent lines that represented a random sample from the set of hybrids used in the advanced trial series of the sorghum pre-breeding program.

### Experimental details

The experiments were conducted at The University of Queensland, St. Lucia, Australia (27°23′S, 153°06′E) in a naturally lit, temperature controlled glasshouse with a day/night temperature regime of 30/22 °C. The BC-NAM population was phenotyped across four independent runs (Table 2) that each consisted of 10 tubs with 50 root chambers each. Experimental runs had two blocks of five tubs and were designed using a row column design where column was represented by the tubs and row by root chamber position within each tub. In each run, 240 genotypes were replicated twice and two check genotypes that were known to differ in root angle (R931945-2-2 and SC-170-6-8, [31]) were each replicated once in each tub. Across runs, the first two had 20% of genotypes in common, whereas run 3 and run 4 had 10% in common (Table 2). This design enabled each pair of runs to be combined in a single analysis.

**Table 2:** Broad sense heritability and genetic correlations for nodal root angle between the experimental runs of the BC-NAM population and the advanced hybrids

Pairs of experiments	Run number	Number of genotypes	Mean root angle (degree)	Heritability (%) (from spatial analysis)	Heritability (%) (from non-spatial analysis)	Percentage of common genotypes (genotypic overlap)	Correlation between runs
BC-NAM population	1 2	199 242	23.6 25.9	77 93	55 89	20	0.94
BC-NAM population	3 4	242 242	26.5 26.0	95 91	85 78	10	0.70
Advanced hybrids	5 6	339 339	27.3 27.2	94 96	93 94	15	0.96

The advanced hybrids were screened in two experimental runs that used a multi-site partially replicated row column design with two experiments that each contained two blocks of five tubs each. Each run included 339 unique hybrids with 50 of these in common across the two experiments, giving a total of 628 hybrids. Within each experimental run, approximately 50% of the hybrids were replicated twice and the other 50% had only a single replicate. The multi-site design enabled the two runs to be combined in a single analysis and replicated hybrids and their randomization to be optimised between the two runs.

### Measurement of nodal root angle

The JPEG files that contained the images of the root systems were used to determine the encompassing angle, relative to the vertical plane, of the first flush of nodal roots. This was done using *openGelPhoto.tcl* (www.activestate.com/activetcl), free software that calculates the angle of individual roots relative to the vertical plane by identifying a point of origin (base of the plant) and an end point for each nodal root. In order to standardize observations, the end point of each root was taken at a distance of 3 cm from the base of the plant. The observed root angle for each plant was the mean of four observations (left and right for both sides of each chamber).The identity of the genotype in each image was tracked using the barcodes that were attached to each individual perspex sheet.

### Statistical analysis

The six experimental runs were analyzed as three independent multi-site trials that consisted of two pairs of runs with sufficient common genotypes to be analyzed together (Table 2). The observed values for nodal root angle (***y***) were analyzed using a linear mixed model, written as

$$\varvec{y} = \varvec{X\tau } + \varvec{Zu} + \varvec{e},$$

where the fixed effect ***τ*** contained the mean root angle in each run and the random effects ***u*** contained genotypes within runs and the genetic correlation between runs. The design matrices for fixed and random effects were given by ***X*** and ***Z*** respectively and ***e*** was the random vector of residual effects. For each run, independent components associated with the structure of the experimental design were included as random effects. These were variation between and within tub.

Possible neighbour effects were allowed by fitting spatial correlations between tubs and root chambers within each run. Since there were 4 measurements within each tub and slot position, an equal variance AR1 model was used for the spatial interaction between tub and slot (AR1v). Despite the relative small footprint of each experimental run, significant spatial effects were detected in all runs and adjustment for these effects generally improved the accuracy of each run by 1–20%. Data were analyzed using ASReml-R [32] and R software Version 3.1.1 (R core team 2014).

In order to quantify the capability of the phenotyping platform to detect genotypic differences in nodal root angle in a repeatable and consistent manner, a broad sense heritability (*H* ^*2*^) was calculated firstly from a model without the spatial AR1v variation and then from a model that included the spatial variation.

## Results

### Genotypic variation for nodal root angle was detected

**Fig. 4:**
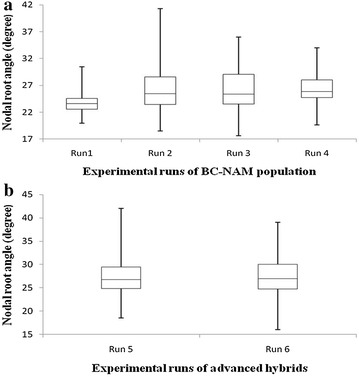
*Box* and *whisker* plots of nodal root angle of sorghum in the phenotyping platform for **a** a BC-NAM population screened across four runs and **b** advanced hybrids screened across two runs. For each run, the bottom and top of the *box* represent the first and third quartile values, the band inside the *box* represents the median value, and the ends of the *whiskers* represent the minimum and maximum values

Genotypic variation in root angle was observed in all experimental runs. Nodal root angle ranged from 17.6° to 41.3° for BC-NAM population and from 16.0° to 42.0° for the advanced hybrids (Fig. 4). Across runs of the BC-NAM population, the minimum root angle ranged from 17.6° (Run 3) to 18.5 (Run 2), whereas the maximum varied more, ranging from 30.5° (Run 1) to 41.3° (Run 2) (Fig. 4a). As a consequence of these differences in maximum nodal root angle, the range in observed phenotypes was lowest in Run 1 (10.5°) and greatest in Run 2 (22.8°). Across the two runs with hybrids, the mean root angle was almost identical (27.3° in Run 5 and 27.2° in Run 6). The greater differences in mean and range in the runs of BC-NAM genotypes was likely due to their increased genetic diversity, because the hybrids represent a random sample from advanced trials that have already undergone selection.

**Fig. 5:**
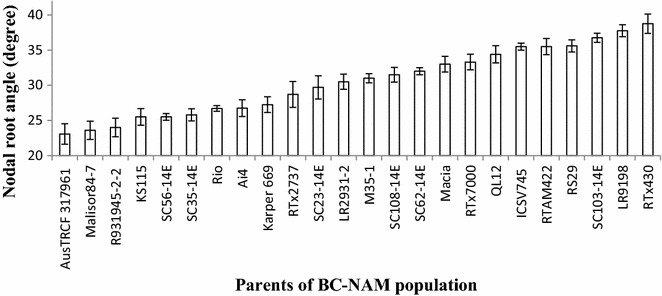
Genetic variation for nodal root angle across 24 parents phenotyped in Run 1 and Run2 of the BC-NAM population. Parents have been sorted for average root angle. The *vertical bars* indicate the relevant standard error

**Fig. 6:**
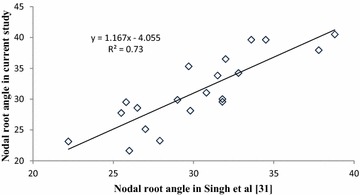
Association between nodal root angle (in degrees) in the current study and that reported by Singh et al. [31] for 20 parents of the BC-NAM population

The nodal root angle of 24 parental genotypes of the BC-NAM population screened in Runs 1 and 2 varied from 23.1° to 38.8° (Fig. 5). Out of the 24 parents, 20 were previously screened for nodal root angle under slightly different experimental conditions using a prototype version of the phenotyping platform used in this study [31]. A strong correlation (*r* ^2^ = 0.73) was observed for nodal root angle recorded for these 20 parents common between both studies (Fig. 6).

### Heritability and genetic correlations for nodal root angle were generally high

High estimates of *H* ^2^ (94 and 96%) for nodal root angle were obtained in both runs of advanced hybrids (Table 2). Without the spatial AR1v effects the *H* ^2^ were 93 and 94% for the advanced hybrids (Table 2). For runs of the BC-NAM population, when spatial effects were included in the analysis *H* ^2^ ranged from 77% (Run 1) to 95% (Run 3) and when spatial effects were not included *H* ^2^ ranged from 55% (Run 1) to 89% (Run 2) (Table 2). These differences in *H* ^2^ estimates of nodal root angle were likely to be a consequence of differences in the genotypes that were included in each run. In general, the high estimates of *H* ^2^ across runs indicated that for individual runs, differences between replications were smaller than those across the genotypes.

Strong genetic correlations were calculated for nodal root angle for all three pairs of experimental runs with common genotypes (Table 2). The genetic correlation between the two advanced hybrid runs was 0.96, whereas for the BC-NAM population it ranged from 0.70 for Runs 3 and 4 to 0.94 for Runs 1 and 2. These high correlations between the three pairs of runs confirmed that the genotypes displayed consistent rankings for nodal root angle across each pair of runs.

### A single experimental run requires around 110 h of user time

The phenotyping platform demonstrated a capacity to identify genetic variation for nodal root angle in large numbers of sorghum lines for a relatively low requirement of user time of 100–120 h per run of 500 plants. Most of this time (nearly 80 h) was required to set up the root chambers, including cleaning and assembly, soil filling, and initial watering. Time requirements for other activities included up to 14 h for sowing, thinning, watering, and application of the nutrient solution and up to 8 h for barcoding, harvesting, and image acquisition. Image analysis and measurement of nodal root angle using the software package takes on average 1 min per image (around 15 h for 1000 images). Hence, the overall time requirement is around 100–120 h, or 13–15 min per plant.

## Discussion

### Late appearance of nodal roots in sorghum requires a soil-based phenotyping platform

Despite the proven utility of nodal root angle in improving drought adaptation, the difficulty of measuring the trait in situ under field conditions has hindered both the genetic dissection and its use as a selection criterion in sorghum breeding programs. Non-soil based phenotyping methods like gel chambers, which have been used in wheat [1, 4], barley [33] and rice [34], and growth pouches and blotting paper, which have been used in maize [27, 35], have been proposed to study root architecture, including root angle, in cereals. These methods were found suitable for root phenotyping in those species, because their root development allowed phenotyping within a few days after germination. Wheat and barley produce multiple seminal roots soon after germination that can be used for phenotyping root angle, whereas in rice and maize, nodal roots appear around the 2nd leaf stage, 1 week after sowing [3, 10]. For sorghum in contrast, the single seminal root and the relatively late appearance of nodal roots [10] require sorghum plants to grow for 3 weeks before root angle can be phenotyped. This can potentially restrict the utility of these non-soil based methods [1, 4, 27, 33–35] that are specifically designed for phenotyping within a few days after germination, before seed reserves used for growth run out.

Nonetheless, non-soil based phenotyping systems that support plant growth for longer periods of time have been developed. Le Marié et al. [36] developed Rhizoslides, a system based on germination paper and plexiglass that was used to phenotype root systems of 20 day old maize plants. Our preliminary studies (Table 1) also indicated that sorghum seedlings can be grown on germination paper and capillary mat for extended periods of time. However, a major problem of the germination paper and capillary mat was that the sorghum roots would penetrate the surface of the media, making acquisition of high quality images cumbersome. It is possible that this behavior of roots might differ across species, because a comparison between maize and sorghum root systems in soil filled rhizotrons suggested that maize and sorghum roots respond differently when hitting the sides of the rhizotrons, with sorghum roots generally growing vertically along the side of the chamber (Fig. 3), whereas maize roots were more likely to reflect off the side back towards the centre of the rhizotrons [10]. This contrasting response could potentially make sorghum roots more prone to penetrating the top layers of the germination paper than maize roots. In addition, the generally poorer growth of sorghum seedlings on germination paper and capillary mat compared to soil-based systems (Table 1) might reflect the high susceptibility of sorghum to water logging during early vegetative stages [37], which could be associated with the lack of aerenchyma in sorghum roots [38]. The specific nature of the root system development of sorghum and its sensitivity to water logging at the seedling stage make the crop not conducive to root phenotyping in non-soil based phenotyping platforms.

Soil-based phenotyping platforms for root architecture traits in general and nodal root angle in particular have been developed before. Richard et al. [39] used 4L transparent pots to image the angle and number of seminal roots in wheat 5 days after sowing. Hargreaves et al. [33] combined soil sacks and X-ray microtomography to measure root traits in barley. However, because of the plant size required for sorghum at the time of imaging and because the root angle observed from 2D gel based phenotyping platform in barley is representative of the 3D angular root spread measured in soil sacks [33], 2D root chambers are logistically the most attractive option. Nagel et al. [40] developed GROWSCREEN-Rhizo, which combined soil-filled rhizotrons for non-destructive 2D measurement of a range of root architecture traits with fully automated imaging at a rate of 60 rhizotrons per hour. Conceptually, this system is quite similar to the system we developed, with the main difference being that our system requires less investment in automating imaging as it requires no sophisticated equipment. This relative simplicity makes our platform adaptable to most glasshouse environments and a version of the system has already been implemented in Africa.

The two critical components of the platform are the root chambers (Fig. 2) and the imaging box (Fig. 3). To facilitate high quality images of roots, it is important to ensure that nodal roots are clearly visible against at least one of the perspex sheets of the root chamber. To achieve this, at sowing the seed needs to be positioned along one of the perspex sheets, because even minor bulging of the perspex sheets can sometimes render the roots invisible against both perspex sheets. Nagel et al. [40] addressed this issue in their setup by having an option to position root chambers at an angle, thus ensuring that a complete root system is visible against the transparent perspex on one side. Apart from the additional costs involved in this feature, the disadvantage compared to our setup is that this will likely result in getting only two estimates for root angle per chamber, whereas our setup generally resulted in four estimates, with increased accuracy and precision. In addition, it is important that the embryo of the seed faces downwards to ensure that the radicle and other roots emerge from the bottom of the seeds and thus avoid gravitropic responses affecting observed root angles. The root chambers also need to be covered to prevent penetration of light to the roots and to prevent algal growth on the perspex sheets. The black polycarbonate sheet we used to cover the top of the tubs that contain the root chambers satisfied these criteria and is readily available in the market. The important elements of the high throughput imaging box were (1) two software controlled digital cameras, (2) four LED light controlling boxes, and (3) two android tablets. The ability to remotely control the digital cameras to capture the image by android tablets through an in-built Wi-Fi^®^ system ensured an image acquisition throughput of at least 60 chambers per hour, similar to the throughput reported by Nagel et al. [40] for GROWSCREEN-Rhizo. The four LED light boxes provided illumination that was uniform across the surface of both perspex sheets for the root chamber inside the imaging box (Fig. 3) and that was consistent across successive root chambers. This uniformity in space and time was key to maintain high resolution of the images obtained. It is likely that our soil-based phenotyping platform can be used for a range of crops. However, for crops for which root angle can be phenotyped within a week from germination, smaller systems that are based on gel, germination paper, or even soil [1, 4, 27, 33–35, 39] might be more appropriate.

The focus during development of our platform was on the phenotyping of nodal root angle. This was guided by observations that this trait is associated with the spatial distribution of roots of mature sorghum plants [7]. This will affect the spatial and temporal ability to extract water from the soil profile [7] and hence grain yield in field conditions [13]. Although other traits associated with root architecture, such as root length, could be measured in our platform, the soil could potentially obscure the fine detail of root architecture required for these measurements (Fig. 3). Although this issue can be resolved by placing the root chambers at an angle [40], the root length density required by sorghum to access all available water is only around 0.2 cm cm^−3^ [41]. Hence, small differences in branching may have only a minor effect on the ability of sorghum plants to extract water. However, branching might be relevant in the context of root system efficiency (RSE), the transpiration per unit leaf area per unit root mass, which represents functional mass allocation to roots to support water capture, relative to allocation to aerial mass that determines water demand [42].

### Phenotypic platform revealed high genetic variation, high heritability, and reproducibility for nodal root angle

The wide range in observed nodal root angle (Fig. 4) and the high heritabilities (Table 2) indicated that the phenotyping platform has the power to detect genetic variation in nodal root angle. The observed range of 17.6°–41.3° (BC-NAM population) and 16.0°–42.0° (advanced hybrids) was comparable with the range of 14.5°–32.3° reported for a recombinant inbred line (RIL) population of sorghum [13]. Variability observed for the parents of the BC-NAM population (23.1°–38.8°) was comparable with that of 21.6°–40.5° in an earlier study [31]. The slightly wider range in nodal root angle observed in the BC-NAM progenies compared to the parental genotypes provided some evidence of minor transgressive segregation for the trait. This can possibly be explained by the recombination of additive alleles of the diverse male parents and a common female line (R931945-2-2), which may have influenced many allelic effects of the female inbred line. The range observed in root angle in the present study was quite different to the range of 60.1°–84.0° and 52.0°–88.0° (relative to the vertical plane) reported for wheat [39] and maize [30] respectively. These species differences were possibly associated with differences in root types (seminal roots for wheat, crown roots for maize, nodal roots for sorghum) and stage of development at the time of phenotyping (seedling stage for wheat, early vegetative for sorghum, mature plants for maize). The overall broad sense heritability *H* ^*2*^ (repeatability) for nodal root angle observed in the present study (91% averaged across the six experimental runs from the spatial analysis and 82.4% averaged from non-spatial analysis, Table 2), was considerably greater than the *H* ^*2*^of 46.6% observed for 44 diverse inbred lines in a previous study [31], but close to the *H* ^*2*^ value of 73.7% reported for a RIL population of sorghum [13]. The high *H* ^*2*^ in our studies indicated that differences in nodal root angle were predominately influenced by genotype, and that variation associated with random factors was minor compared to the genetic variation. The magnitude of the heritability for a trait measured in a phenotyping platform is an important factor in determining its efficiency and relevance to a large scale germplasm screening programs.

The accuracy and repeatability of the phenotyping platform was further highlighted by strong genetic correlations for the three pairs of experimental runs (Table 2) and by the high correlation with the results reported by Singh et al. [31] under different experimental conditions (Fig. 6). Overall, strong genetic correlations, high repeatability (heritability) and consistent ranking obtained across runs in different sets of genetic material indicate that the platform could provide a useful screen for nodal root angle across the diverse germplasm of a breeding program. This would make root angle an important selection trait in breeding programs that aim at improving drought adaptation of sorghum through genetic manipulation of RSA.

### Value of phenotyping for nodal root angle to breeding programs

Higher efficiency in selecting for RSA can be achieved by designing phenotyping platforms that are capable of (1) screening root traits at the seedling stage to shorten the selection cycle and speed up the rate of genetic improvement and (2) establishing the genetic correlation between the root trait phenotyped and the ultimate breeding target (grain yield) [43]. The advanced hybrids screened for nodal root angle in the current study were also evaluated for grain yield in seven different environments across Australia. Analysis of the data revealed an association between narrow nodal root angle and grain yield across environments (D Jordan, unpublished data). This was consistent with the findings of Mace et al. [13], who reported a weak but significant association between the presence of QTL for narrow root angle and grain yield in a set of RILs of sorghum evaluated in hybrid combination in yield trials. This association reflects the observation that in sorghum, QTL for nodal root angle co-locate with QTL for stay-green [13], the ability of a crop to retain green leaf area during grain filling [44]. A possible mechanism for this would be that narrow root angle could increase the ability of plants to access water from deeper soil layers [7, 45], which can prolong maintenance of photosynthesis and remobilization activities during grain filling [44] under drought. In this context, it is interesting to note that genotypes SC56-14E, SC35-14E, Rio, and R931945-2-2, which are the main sources of stay green in various breeding program across the world [23], each had narrow nodal root angle (Fig. 5). Moreover, four near isogenic lines (NILs) that each contained a single introgression of a stay green QTL (NIL6078-1, *Stg1*; NIL2219-3, *Stg2*; NIL2290-19, *Stg3*;NIL6085–9, *Stg4*) displayed narrow root angle as well (data not shown). These results indicate that the sorghum breeding program in Australia has likely indirectly selected for narrow root angle when pursuing improved adaptation to post- flowering drought stress as one of its most important breeding objectives. Hence, nodal root angle at the seeding stage can be predictive of grain yield across environments.

## Conclusion

The platform presented in this paper provides a high throughput, low cost, easy to implement screen for phenotyping nodal root angle of sorghum. The setup requires no sophisticated instruments, has a relatively small foot print and allows rapid, non-destructive, two dimensional analysis of root angle with minimal disturbance to plant growth. Integration of the high throughput phenotyping platform with advanced genomic approaches allows identification of QTLs governing nodal root angle and mining of alleles to tailor RSA of genotypes to predominant management and environmental conditions to exploit specific adaption to drought stress. Apart from screening large breeding populations for genetic mapping, this platform is equally applicable to enhancing the efficiency of breeding programs. For instance, high throughput screening of elite inbred lines and a large number of experimental hybrids at early seedling stage will assist in their selection, release and adoption for a particular production environment (terminal drought stress environment, skip row management system). In addition, rapid screening of early segregating generations (F_2_ and F_3_) will enhance the selection efficiency and enrich the gene pool with the alleles governing desirable root phenotype (narrow or wide angle). The platform would also be suitable to evaluate the post-embryonic root architecture of any other graminaceous species for which nodal roots appear at similar development stages as sorghum.
